# The Utility of Monitoring Beta-Human Chorionic Gonadotropin Levels in an Ectopic Pregnancy

**DOI:** 10.7759/cureus.34063

**Published:** 2023-01-22

**Authors:** LauraAnne E Hirschler, Varun Soti

**Affiliations:** 1 Obstetrics and Gynecology, Lake Erie College of Osteopathic Medicine, Elmira, USA; 2 Pharmacology and Therapeutics, Lake Erie College of Osteopathic Medicine, Elmira, USA

**Keywords:** tubal ectopic pregnancy, pregnancy surveillance, transvaginal ultrasound, beta-human chorionic gonadotropin (β-hcg), ectopic pregnancy

## Abstract

Ectopic pregnancy, a rare complication involving embryo nidation outside the uterus, significantly impacts women's lives worldwide. About 95% of ectopic pregnancies occur in the Fallopian tubes. If not diagnosed early, the patient may suffer from tubal rupture, resulting in hemorrhage and lethal consequences. Transvaginal ultrasound (TVUS) is typically used to diagnose an ectopic pregnancy. However, over the last decade, monitoring beta-human chorionic gonadotropin (β-hCG) levels in ectopic pregnancy have evolved to detect ectopic pregnancy. But there are inconsistencies in its utility in monitoring or diagnosing ectopic pregnancy in clinical practice. This systematic review highlights the potential of monitoring β-hCG levels to accurately diagnose ectopic pregnancy. Furthermore, it showcases if β-hCG levels can determine effective treatment options to successfully resolve an ectopic pregnancy. We performed a literature search between January 2022 through December 2022 following the Preferred Reporting Items for Systematic reviews and Meta-Analyses (PRISMA) guidelines. The clinical evidence demonstrated that monitoring β-hCG levels, combined with TVUS, accurately diagnosed an ectopic pregnancy. Moreover, pre-treatment β-hCG levels higher than 5000 international units per liter (IU/L), statistically significant, indicated surgical management for a successful resolution of an ectopic pregnancy. Whereas lower β-hCG levels showed successful management through expectant and methotrexate treatment. Interestingly, patients who failed non-surgical treatment developed increased β-hCG levels and required surgical intervention. However, there was conflicting evidence on whether β-hCG levels could indicate tubal rupture. Nevertheless, as highlighted in this review, monitoring β-hCG levels could be crucial in the early diagnosis of ectopic pregnancy. Besides, it might significantly aid in monitoring and deciding on effective treatment options for patients with ectopic pregnancy, which could be vital to saving their lives and preserving fertility.

## Introduction and background

Although a rare pregnancy complication, ectopic pregnancy significantly impacts the lives of women, their children, and families worldwide. Every woman’s experience with ectopic pregnancy is different, calling for the need to individualize care plans and management techniques [1]. Ectopic pregnancies can occur in various locations, but the most common of these locations are tubal-ectopic pregnancies involving the Fallopian tubes accounting for 95% of ectopic pregnancies [2].

Tubal-ectopic pregnancies account for the most significant maternal deaths during the first trimester of pregnancy. With advancements in sonography, early diagnosis and initiation of care are implemented in patients suffering from tubal-ectopic pregnancy. An obstetrician/gynecologist must consider every aspect of a patient when developing a management plan for tubal-ectopic pregnancy. It is critical to diagnose an ectopic pregnancy quickly and accurately before it ruptures [1].

Monitoring levels of beta-human chorionic gonadotropin (β-hCG) [3] as well as transvaginal ultrasound (TVUS) [4] investigation allows for accurate diagnostics to determine tubal-ectopic pregnancies [1]. The TVUS allows for the visualization of the ectopic pregnancy and provides the ability to perform a visually-guided bimanual examination. TVUS accounts for 90.9% of successful diagnoses of ectopic pregnancies [5]. In addition, the utilization of TVUS has allowed for better management of ectopic pregnancy, leading to a drastic decrease in the dilatation and curettage procedure [6].

When evaluating patients with ectopic pregnancy, it is crucial to analyze the entire abdominal cavity and the pelvis to assess for rupture. TVUS can be coupled with a suprapubic examination to evaluate the possibility of an ectopic pregnancy. It can identify intrauterine pregnancies after 42 days of gestation. β-hCG is a valuable biomarker for the early prediction of ectopic pregnancy. Its elevated levels outside the normal range indicate the presence of ectopic pregnancy [7].

In addition, β-hCG is reliable in assessing and determining the severity of ectopic pregnancies. Therefore, measuring serum β-hCG levels can help effectively formulate treatment plans to resolve an ectopic pregnancy. Typically, β-hCG levels in non-pregnant, pre-menopausal women range from 0.02-0.8 international units per liter (IU/L). However, in normal intrauterine pregnancies detected via ultrasound, β-hCG levels are 1000 IU/L or higher. They rapidly rise and peak by the 10th week of gestation, typically between 20000 and 200000 IU/L. After ten weeks, β-hCG levels may decline or remain steady [8].

It is worth understanding the ‘discriminatory level’ of β-hCG, which is crucial in diagnosing an ectopic pregnancy. The discriminatory level refers to the concentration of β-hCG at which TVUS sensitivity detects an intrauterine pregnancy [1]. However, if it is an ectopic pregnancy, β-hCG levels rise above the discriminatory level. Various levels have been used as the discriminatory level of β-hCG, including 1000 IU/L, 1500 IU/L, and 2000 IU/L [9]. Some researchers have demonstrated that using the discriminatory level of β-hCG to diagnose ectopic pregnancy is not entirely reliable [10-12]. Instead, using serial β-hCG levels can diagnose an ectopic pregnancy more accurately. Serial β-hCG levels obtained 48 hours apart if they are below optimal rise than expected in an intrauterine pregnancy or fall in titers less than in a spontaneous miscarriage indicate an ectopic pregnancy and help in its accurate diagnosis [13].

Therefore, β-hCG is a reliable biomarker aiding in diagnosing ectopic pregnancies. When combined with TVUS, measuring β-hCG levels can be helpful in an early diagnosis of an ectopic pregnancy. An easily measured and accurate means of diagnosing an ectopic pregnancy through a simple biomarker such as β-hCG would enhance successful and life-saving management of ectopic pregnancies.

Despite the growing consensus among obstetricians and gynecologists on the utility of monitoring β-hCG in ectopic pregnancies, there has been inconsistency in its use in clinical practice as an effective screening or diagnostic tool. Moreover, β-hCG's role and effectiveness in serving as the aid in deciding on treatment and management of patients with ectopic pregnancy is not well-defined.

Therefore, the review aims to determine if monitoring β-hCG levels can accurately aid in an early diagnosis of an ectopic pregnancy. Furthermore, it seeks to identify if β-hCG estimates can determine effective pharmacological and surgical treatment strategies to resolve ectopic pregnancy successfully.

## Review

Literature search and study selection

We conducted a literature search from January 2022 to December 2022 following Preferred Reporting Items for Systematic Reviews and Meta-Analyses (PRISMA) guidelines [14]. We searched three databases: PubMed, BioMed Central, and EBSCO. The keywords used in the literature search were “Ectopic Pregnancy + β-hCG,” “β-hCG,” “Ectopic Pregnancy + Diagnosis,” “β-hCG, Pregnancy,” and “β-hCG + Tubal Pregnancy.” The studies included were clinical (randomized clinical trials, non-randomized, blinded, double-blinded, and pilot), retrospective, observational, and cross-sectional. The select patient demographics were women in the reproductive age group and below 50. We selected relevant studies written in the English language that met the inclusion criteria (Table 1). The literature search was not limited to studies published in the last 10 years, and as such there was no set year limit.

**Table 1:** Study Selection Criteria. Studies published in English, focusing on patients with select demographics (women of reproductive age below 50, with patent ectopic pregnancy or tubal rupture), and meeting the inclusion criteria, were included in the review.

Inclusion Criteria	Exclusion Criteria
Randomized clinical trials	Metanalysis
Non-randomized clinical trials	Reviews
Blinded and non-blinded clinical studies	
Pilot clinical studies	
Observational clinical studies	
Cross-sectional clinical studies	
Retrospective analysis	

**Figure 1:**
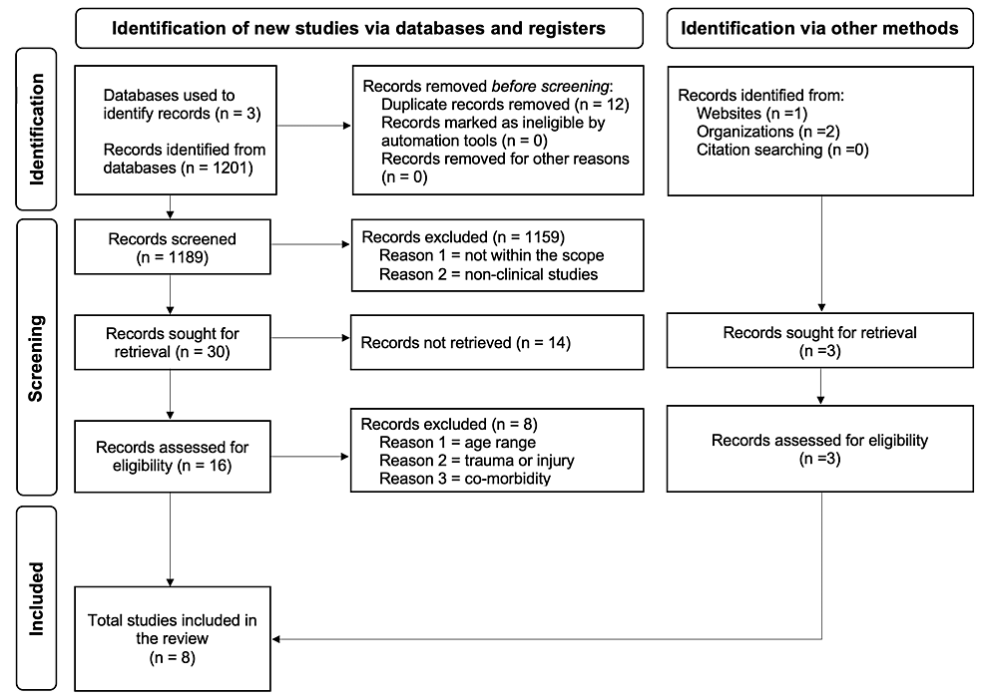
Literature Search and Study Selection Process. This systematic review followed the Preferred Reporting Items for Systematic Reviews and Meta-Analyses guidelines [14] and utilized PubMed, BioMed Central, and EBSCO to search for clinical studies on beta-human chorionic gonadotropin (β-hCG) monitoring in patients with ectopic pregnancy. First, the keywords were limited to “Ectopic pregnancy + β-hCG,” “β-hCG,” “β-hCG, Pregnancy,” and “Ectopic Pregnancy + Diagnosis.” Then, by using filters and inclusion criteria, including articles written in English, complete clinical studies, retrospective clinical studies, and clinical observational and cross-sectional studies, with select patient demographics (patients in the reproductive age group and below 50, patients with patent ectopic pregnancy and tubal rupture), we selected relevant studies. Moreover, the literature search was not limited to studies published in the last 10 years, and as such there was no set year limit.

Per the previous literature [15], we assigned a level of clinical evidence to the studies included in this review. See Figure 1for the literature search and selection process.

Ectopic pregnancy

Ectopic pregnancy is the presence of a gestational sac implanted outside the uterus. In women within the reproductive age range, the incidence of having an ectopic pregnancy has been steadily increasing in the United States [16]. Common ectopic pregnancy implantation sites include the fallopian tubes, cornua of the uterus, abdominal cavity, cervix, and ovaries [17]. The most common form of ectopic pregnancy is tubal ectopic pregnancy, comprising 95% of all ectopic pregnancies. About 2.5% occur in the cornua of the uterus, and the abdominal cavity, cervix, and ovaries account for 1.5% of ectopic pregnancies [18]. Further, with rates of cesarean sections increasing, it has been reported that the scar left behind from the hysterotomy can serve as a possible implantation site, accounting for 1% of ectopic pregnancies [19].

Risk factors of ectopic pregnancy

In addition to cesarean sections serving as an ectopic pregnancy risk factor, numerous other risk factors exist. These include a history of previous ectopic pregnancy, the presence of an intrauterine device at conception, assisted reproductive technique usage, pelvic inflammatory disease, and smoking. Also, the number of times a woman has been pregnant is a predisposing risk factor for an ectopic pregnancy. It has been estimated that multigravida women account for two-thirds of ectopic pregnancies [20]. Therefore, it is of the utmost importance to accurately and quickly diagnose an ectopic pregnancy. If left undiagnosed, ectopic pregnancies can become a grave emergency putting the patient at risk for intraperitoneal bleeding and tubal rupture. Substantial morbidity and the possibility of mortality are faced in ectopic pregnancy diagnoses that are complex and more severe [20].

Clinical manifestations of ectopic pregnancy

An ectopic pregnancy may remain asymptomatic until pelvic fluid, a fetal pole with a yolk sac, and a gestational sac begins to increase in size. Signs of a tubal rupture include abdominal and shoulder pain, peritoneal irritation, and hemodynamic instability, including hypovolemic shock and hemorrhage [21]. Hypovolemic shock and hemorrhage represent up to 10% of patients with ectopic pregnancies and contribute to a 9% mortality rate [22]. Additionally, patient fertility may be impacted in these cases. Therefore, women experiencing abdominal pain and/or bleeding coupled with amenorrhea during their reproductive years must be considered to rule out a diagnosis of ectopic pregnancy [21].

Current diagnostic practice for ectopic pregnancy

Although β-hCG is used to determine whether or not a woman is pregnant, its measurement is not considered the sole standard diagnostic procedure for ectopic pregnancy. Currently, TVUS is used to diagnose ectopic pregnancy. About 85% of ectopic pregnancies are detected before the tubal rupture [22]. With the use of doppler ultrasound, a sensitivity of about 90% can be achieved when the "ring of a fire" sign is observed surrounding the mass in the adnexa, indicating ectopic pregnancy. Should a diagnosis of ectopic pregnancy be missed, the patient risks hypovolemic shock, rupture of her tubes, salpingectomy, and blood transfusions, increasing morbidity [22].

Current management of ectopic pregnancy

With enhanced TVUS quality, awareness early in the course of an ectopic pregnancy, and heightened serum β-hCG sensitivity, the management strategies have shifted to preserving fertility and the Fallopian tubes. When an ectopic tubal pregnancy is detected early on, the necessity for laparotomy is diminished, and less invasive treatment options are utilized. Treating an ectopic pregnancy medically through administering methotrexate, performing laparoscopic surgery conservatively, and managing the pregnancy expectantly are all practical approaches depending on each patient’s unique case [22]. Surgical options such as laparoscopic salpingectomy and laparoscopic salpingostomy are commonly utilized to treat and manage ectopic pregnancies regardless of the rupture status. It is advisable to treat with methotrexate if selection criteria are fulfilled, such as a gestational sac that is small and intact, asymptotic patient presentation, the absence of embryonic cardiac activity, and low levels of β-hCG [21-22]. Traditionally, ectopic pregnancies are managed via salpingostomy procedures. However, if diagnosed early enough, methotrexate can be used as a conservative management approach, a method whose efficacy is most significant during the early stages of an ectopic pregnancy diagnosis [22-23]. Methotrexate exerts its effect by inhibiting cell division and competitively inhibiting folic acid [23].

Clinical evidence of the utility of monitoring β-hCG levels in ectopic pregnancy

Al Sayed (2011) [24] was one of the few earliest researchers who investigated whether β-hCG could correctly predict ectopic pregnancy. In a prospective study enrolling 160 patients, he aimed to assess β-hCG’s role as a predictor of tubal patency in patients who had received conservative treatment for ectopic pregnancies. After the study subjects underwent TVUS-guided selective salpingography to confirm the ectopic pregnancy, 60 received a single dose of methotrexate, and 100 received expectant management. All patients were submitted to two quantitative measurements of β-hCG at 24/48-hour intervals, and the enzyme immunoassay method was utilized to measure serum β-hCG. The findings showed β-hCG was the factor that significantly indicated the increased risk of tubal obstruction after the clinical treatment of ectopic pregnancy. The serum levels of β-hCG were higher than 5000 IU/L with an odds ratio of 11.7 (p = 0.0033) [24].

Furthermore, the logistic regression of the study data demonstrated that β-hCG levels were directly associated with the risk of tubal obstruction [24]. In other words, an increase in β-hCG levels led to a substantial augmentation in ipsilateral tube obstruction risk. Interestingly, no other factors directly contributed to or indicated an increased risk of tubal obstruction. The researcher concluded that β-hCG levels influenced patients’ chances of having ipsilateral fallopian tube obstruction due to ectopic pregnancy. A greater risk of obstruction was associated with elevated β-hCG levels [24]. Therefore, β-hCG could be a valuable marker not only in predicting ectopic pregnancy but also in protecting the patient’s fertility, and hence, it could be a tremendous tool to successfully assess ectopic pregnancy in routine clinical practice.

The emerging perception that β-hCG levels could help correctly diagnose patients with ectopic pregnancy and indicate its successful resolution was broadened and validated by Mavrelos et al. (2015) [25]. They retrospectively analyzed data from 474 patients diagnosed with ectopic pregnancy utilizing TVUS. Their study revealed that patients with β-hCG levels of 1500 IU/L or higher needed surgical intervention to successfully resolve ectopic pregnancy. However, 158 patients out of 226, approximately 70% of the sample, had β-hCG HCG levels under 1500 IU/L and required expectant management to treat the ectopic effectively, which was statistically significant at p < 0.05 [25]. Interestingly, the successful resolution of ectopic pregnancy over three weeks was directly linked with a gradual decline in β-hCG levels, with the lowest levels of less than 20 IU/L at the end of the treatment. In addition, the study showed that 18 days was the median interval from the highest serum β-hCG concentration measured to the resolution of the ectopic pregnancy, showcasing measuring β-hCG levels was critical to monitoring the ectopic pregnancy and successfully resolving it [25].

These researchers [25] demonstrated that patients’ initial levels of β-hCG at the time of diagnosis were positively associated with ectopic pregnancy, the type of treatment course taken to resolve ectopic pregnancy safely and effectively, and follow-up lengths of under three weeks in women with ectopic pregnancies who had expectant management. This study showcased that β-hCG levels could accurately diagnose patients with ectopic pregnancy and suggested their potential in effectively monitoring the patient and determining the appropriate course of treatment [25]. 

The future potential of β-hCG levels in diagnosing and managing patients with ectopic pregnancy prompted more research endeavors. One such crucial effort was made by Orozco et al. (2015) [26]. In a prospective observational study, they investigated the accuracy of β-hCG levels measurement in determining the success of medical treatment (methotrexate) in patients with ectopic pregnancy. The study enrolled patients with confirmed ectopic pregnancy diagnosis by TVUS and β-hCG levels under 5000 IU/L. It required measuring β-hCG levels on days zero, four, and five of the methotrexate treatment. Of the 126 patients who met the study criteria, 111 (88%) had successful methotrexate treatment; and of the remaining 15 (12%), 11 needed surgery due to ruptured ectopic, and four had abdominal pain and, therefore, underwent surgery [26].

The study findings further showed that patients who had successful methotrexate treatment had median β-hCG levels of 1.482 ± 950 on day zero, 1.469 ± 1314 on day four, and 1.092 ± 1147 on day seven compared to those who failed methotrexate treatment, with their β-hCG levels of 2.166 ± 816 on day zero (p < 0.03), 3.595 ± 2.115 on day four (p < 0.001), and 3.663 ± 2.375 on day seven (p < 0.001), respectively. It was noteworthy that apart from β-hCG levels, no other factors, including patients’ age, nulliparity, adnexal mass size, and ectopic antecedent, could predict treatment success rate [26].

Orozco et al. (2015) [26] evidenced the reliability of using β-hCG levels measurement in determining ectopic pregnancy. In addition, they demonstrated their accuracy in predicting the success rate of medical treatment rendered to patients with ectopic pregnancy. These results reflected β-hCG in a different light. They showcased measuring β-hCG levels as an accurate diagnostic tool in correctly diagnosing ectopic pregnancy and effectively predicting effective treatment outcomes in such patients.

Shiravani et al. (2022) [27] further substantiated the utility of measuring β-hCG levels in accurately diagnosing patients with ectopic pregnancy and positively predicting the success of treatment methods used to manage such patients. In a cross-sectional study, they analyzed data from 365 patients diagnosed with ectopic pregnancy. Of 365, 154 underwent surgery, 87 received single-dose methotrexate treatment, 43 received double-dose methotrexate, and 81 had expectant treatment [27].

The study results showed that β-hCG levels (more than 6419 IU/L) predicted surgery as the best treatment plan with 47% sensitivity and 90% specificity (p < 0.01). Moreover, β-hCG levels also indicated the successful treatment with single-dose methotrexate treatment. Patients who had successful resolution of ectopic pregnancy with single-dose methotrexate had β-hCG levels of 1289.44 ± 1433.29 IU/L (p < 0.02) than those who failed treatment had β-hCG levels of 8110.21 ± 10060.76 IU/L [27]. These findings further strengthened the reliability of using β-hCG levels in diagnosing ectopic pregnancy and determining the successful treatment options, surgical and non-surgical (methotrexate), for best patient outcomes.

In yet another study, Surampudi & Gundabatulla (2016) [3] evaluated trends of β-hCG levels in determining ectopic pregnancy management. They retrospectively analyzed data from 337 patients with ectopic pregnancies. The study findings showed the average initial (pre-treatment) β-hCG levels were 3866.20 IU/L in those who received treatment with methotrexate (single dose or double dose of methotrexate with or without mifepristone) compared to 12961.50 IU/L in those who underwent surgical intervention. Interestingly, those who received methotrexate treatment and successfully resolved ectopic pregnancy reported 3351.10 IU/L as the average initial β-hCG level compared to 5730.00 IU/L in patients who failed the methotrexate treatment and subsequently underwent surgery [3]. However, the authors did not document specific trends in the rapid or steady rise or fall in β-hCG levels post-treatment and did not statistically analyze their findings.

Conversely, there was conflicting evidence regarding the effectiveness of using β-hCG levels measurements in predicting ruptured ectopic pregnancy. In a retrospective observational analysis, Li et al. (2022) [28] investigated data from 225 patients diagnosed with ectopic pregnancy. They aimed to assess clinical characteristics and risk factors that could indicate the prognosis of ruptured ectopic pregnancy. Of 225 patients, 176 suffered from a ruptured ectopic pregnancy, and 49 had patent ectopic pregnancy. In patients who had ruptured ectopic pregnancy, study results demonstrated that abdominal pain was higher (p < 0.01), pre-operative hemoglobin was lower (p < 0.001), and blood loss and blood transfusion were higher with p = 0.000 and p = 0.001, respectively compared to those who had patent ectopic pregnancy. Most notably, there was no remarkable difference between pre-treatment β-hCG levels (p = 0.3) in the two groups. In addition, factors such as ectopic pregnancy history, abdominal surgeries, and tubal surgeries did not significantly impact the patients who suffered ruptured ectopic pregnancy [28]. The study findings showed that β-hCG levels did not predict ruptured ectopic pregnancies.

However, Cohen et al. (2022) [29] demonstrated how changes in β-hCG levels accurately predicted if the ectopic pregnancy would rupture or remain patent. In a retrospective study, they analyzed data from 401 patients diagnosed with ectopic pregnancy. Their analysis showed that of 122 patients who received methotrexate treatment, 41 had ruptured ectopic pregnancy. The median time interval from day zero of methotrexate to tubal rupture was six days. Notably, the β-hCG level percentage change in 48 hours before methotrexate treatment predicted tubal rupture. For every change in the β-hCG level, the odds ratio was 1.08 with a 95% confidence interval of 1.04 - 1.12 (p < 0.001). The study outcomes revealed that patients who had an increment in β-hCG levels by more than 69% 48 hours before methotrexate treatment had an 85% probability of tubal rupture. Moreover, patients with a β-hCG level increment of less than 20% 48 hours before methotrexate treatment had a low risk of absolute tubal rupture [29]. The researchers showcased that β-hCG levels could indicate the probability of tubal rupture and recommended that physicians assess its risk before starting a patient on methotrexate treatment.

The outcomes of the studies conducted by Li et al. (2022) [28] and Cohen et al. (2022) [29] were contradictory. With insufficient evidence, it was challenging to postulate the precise role of β-hCG levels in ruptured ectopic pregnancy. Nevertheless, these findings created an opportunity for others to further their research efforts in shedding light on the part of β-hCG levels in indicating ruptured ectopic pregnancy.

In addition to using β-hCG levels in diagnosing ectopic pregnancy (patent and/or ruptured), researchers, such as Al Niami et al. (2021) [30], in a single-center retrospective analysis, highlighted that β-hCG levels could indicate ectopic pregnancy-related morbidity. Examining the data from 30,247 pregnant patients, they found that patients with ectopic pregnancies had an average β-hCG level of 5509 ± 4131 IU/L and predicted morbidity associated with ectopic pregnancy with a β-coefficient of 0.01 and a 95% confidence interval of 0.0006 - 0.022 (p < 0.04) [30].

See Table 2** **for key studies investigating the clinical utility of measuring β-hCG levels in ectopic pregnancy.

**Table 2:** Key Studies evaluating the monitoring of beta-human chorionic gonadotropin (β-hCG) levels in accurately predicting ectopic pregnancy. The studies meeting the inclusion criteria for this review, including clinical prospective, retrospective, cross-sectional, and observational studies, demonstrated that β-hCG levels accurately predicted ectopic pregnancy and indicated related morbidity. Moreover, monitoring β-hCG levels significantly led to the best treatment course for effective resolution of ectopic pregnancy. *IU/L*, International Units per Liter; *p*, Probability Variable.

Authors	Level of Evidence	Type of Study	Sample Size	Study findings
Al Sayed (2011) [24]	II.1	Prospective	160 patients	β-hCG levels positively predicted ectopic pregnancy. β-hCG levels higher than 5000 IU/L indicated significant risk of tubal obstruction (p = 0.0033).
Mavrelos et al. (2015) [25]	II.1	Retrospective	474 patients	Monitoring β-hCG levels helped predict ectopic pregnancy. Moreover, β-hCG estimates effectively determined a treatment course to manage ectopic pregnancy. β-hCG levels higher than 1500 IU/L led to successful resolution of ectopic pregnancy via surgical methods. Whereas β-hCG levels lower than 1500 IU/L necessitated expectant treatment to effectively resolve ectopic pregnancy (p < 0.05).
Orozco et al. (2015) [26]	II.1	Prospective	126 patients	Monitoring β-hCG levels accurately determined the success of medical treatment with Methotrexate in patients with ectopic pregnancy. Patients with successful Methotrexate treatment had median β-hCG levels of 1.482 ± 950 on day zero, 1.469 ± 1314 on day four, and 1.092 ± 1147 on day seven compared to those who failed methotrexate treatment, with their β-hCG levels of 2.166 ± 816 on day zero (p < 0.03), 3.595 ± 2.115 on day four (p < 0.001), and 3.663 ± 2.375 on day seven (p < 0.001), respectively.
Shiravani et al. (2022) [27]	II.1	Cross-sectional	365 patients	β-hCG levels of more than 6419 IU/L predicted surgery as the best treatment plan with 47% sensitivity and 90% specificity (p < 0.01). β-hCG levels also indicated the successful treatment with single-dose Methotrexate treatment. Patients who had successful resolution of ectopic pregnancy with single-dose Methotrexate had β-hCG levels of 1289.44 ± 1433.29 IU/L (p < 0.02) than those who failed treatment had β-hCG levels of 8110.21 ± 10060.76 IU/L.
Surampudi & Gundabatulla (2016) [3]	II.1	Retrospective	337 patients	Initial β-hCG levels indicated the best treatment option to effectively resolve ectopic pregnancy. Patients with an average initial β-hCG level of 3351.10 IU/L had successful treatment with Methotrexate. Whereas patients with higher β-hCG levels successfully resolved ectopic pregnancy through surgery. However, researchers did not perform statistical analysis.
Li et al. (2022) [28]	II.1	Observational	225 patients	Initial β-hCG levels did not indicate if the ectopic pregnancy would be patent or ruptured. There was no significant difference in pre-treatment β-hCG levels in the study patients with patent ectopic pregnancy than those with tubal rupture (p = 0.3).
Cohen et al. (2022) [29]	II.1	Retrospective	122 patients	Changes in β-hCG levels accurately predicted if the ectopic pregnancy would rupture or remain patent. The β-hCG level percentage change in 48 hours before Methotrexate treatment predicted tubal rupture. For every change in the β-hCG level, the odds ratio was 1.08 with a 95% confidence interval of 1.04 – 1.12 (p < 0.001). Patients with an increment in β-hCG levels by more than 69% 48 hours before Methotrexate treatment had an 85% probability of tubal rupture. Moreover, patients with a β-hCG level increment of less than 20% 48 hours before Methotrexate treatment had a low risk of absolute tubal rupture.
Al Niami et al. (2021) [30]	II.1	Retrospective	30247 patients	β-hCG levels indicated ectopic pregnancy-related morbidity. Patients with ectopic pregnancies had an average β-hCG level of 5509 ± 4131 IU/L and predicted morbidity associated with ectopic pregnancy with a β-coefficient of 0.01 and a 95% confidence interval of 0.0006 – 0.022 (p < 0.04).

## Conclusions

This review presents literature on the utility of monitoring β-hCG levels in patients with ectopic pregnancy. The existing clinical evidence indicates β-hCG estimates, when combined with TVUS, can help in early and accurate diagnosis of ectopic pregnancy. It further demonstrates that measuring β-hCG levels can be a crucial tool for obstetricians/gynecologists to make the right treatment decision regarding opting for surgical options and traditional management strategies: expectant and methotrexate treatment. The current clinical literature suggests that early detection of ectopic pregnancy through non-invasive and routine monitoring of β-hCG levels will result in the best patient outcomes in resolving ectopic pregnancy and decrease patient mortality and morbidity, contributing toward preserving patient fertility. However, there are contradictory and insufficient data on whether β-hCG levels can accurately predict tubal rupture. Therefore, further concerted research efforts are required to investigate the role of β-hCG in tubal rupture and how it can be therapeutically targeted to treat such patients in the future. 
